# The value of gadobenate dimeglumine-enhanced MRI quantification in predicting aggressiveness and prognosis of typical intrahepatic mass-forming cholangiocarcinoma: a multicenter retrospective study

**DOI:** 10.1186/s13244-026-02225-4

**Published:** 2026-03-03

**Authors:** Shuo Zhang, Bing Kang, Chenyang Qiu, Kai Deng, Haitao Sun, Yicong Nie, Ximing Wang, Cong Sun

**Affiliations:** 1 Department of Radiology, Shandong Provincial Hospital Affiliated to Shandong First Medical University, Jinan, China; 2 Department of Radiology, The First Affiliated Hospital of Shandong First Medical University, Jinan, China; 3 Medical Imaging Center, Central Hospital Affiliated to Shandong First Medical University, Jinan, China; 4 Department of Pathology, The First Affiliated Hospital of Shandong First Medical University, Jinan, China

**Keywords:** Intrahepatic mass-forming cholangiocarcinoma, Magnetic resonance imaging, Hepatobiliary phase, Overall survival

## Abstract

**Objective:**

This study aimed to evaluate the predictive value of quantitative gadobenate dimeglumine-enhanced MRI parameters in aggressiveness and prognosis of intrahepatic mass-forming cholangiocarcinoma (IMCC).

**Materials and methods:**

A total of 158 patients with IMCC who underwent preoperative MRI at three centers were included, and their clinical and imaging data were analyzed retrospectively. Multimodal quantitative parameters were measured in various tumor areas, including relative intensity ratio (RIR) and relative enhancement ratio (RER) of the central and rim areas of the tumor to the liver in the hepatobiliary phase, and the center area-tumor volume ratio. Patients were classified into low-aggressiveness (Ki-67 LI < 25%) and high-aggressiveness (Ki-67 LI ≥ 25%) groups based on the Ki-67 labeling index (LI). Potential risk factors of aggressiveness were determined using multivariate logistic regression analysis. The prediction efficacy of factors was assessed using receiver operating characteristic (ROC) curves. Overall survival (OS) and disease-free survival (DFS) were evaluated using the Cox proportional-hazards regression model.

**Results:**

The volume ratio (VR) and RIR_rim_ were independent risk factors for aggressiveness (*p* < 0.05). The area under the ROC curve was 0.803 [95% confidence interval (CI), 0.728–0.878] and 0.799 (95% CI, 0.727–0.872), both higher than that of CA19-9 ≥ 34 U/mL and intratumoral necrosis (all, *p* < 0.05). VR and RIR_rim_ were identified as independent predictors of OS and DFS in patients with IMCC (*p* < 0.05).

**Conclusion:**

The multimodal quantitative MRI parameters, VR and RIR_rim_, were effective risk factors for predicting both aggressiveness and prognoses in patients with IMCC.

**Critical relevance statement:**

Noninvasive MRI hepatobiliary-phase quantification stratified aggressiveness and prognosis in intrahepatic mass-forming cholangiocarcinoma. It might provide important clinical information for treatment strategies.

**Key Points:**

- The volume ratio (VR), relative intensity ratio (RIR_rim_), CA19-9 ≥ 34 U/mL, and necrosis were independent predictors of high aggressiveness.
- The VR, RIR_rim_, CA19-9 ≥ 34 U/mL, and tumor boundary were independent predictors of poorer overall survival.
- The VR, RIR_rim_, CA19-9 ≥ 34 U/mL, tumor boundary, and tumor maximum size ≥ 3 cm were independent predictors of shorter disease-free survival.

**Graphical Abstract:**

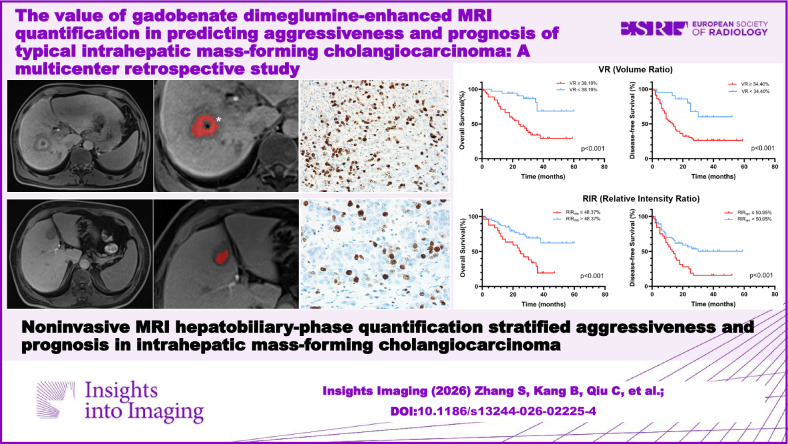

## Introduction

Intrahepatic cholangiocarcinoma is the second most common intrahepatic primary malignancy [1, 2]. The Japanese liver cancer research group has classified tumor growth in intrahepatic cholangiocarcinoma into mass-forming, periductal infiltrative, and intraductal [3]. The intrahepatic mass-forming cholangiocarcinoma (IMCC) is the most common, accounting for approximately 78% of cases [4]. Surgery is considered an effective treatment; however, the 5-year overall survival (OS) and the postoperative recurrence rate after surgery are 20–35% and 50–70%, respectively [5].

The Ki-67 protein is a nuclear antigen related to cell proliferation and is positively associated with cancer aggressiveness [6]. In a clinical context, the cell proliferation Ki-67 labeling index (LI) is often used to assess tumor aggressiveness [7]. Qiang et al [8] demonstrated an association of a higher Ki-67 status with advanced tumor-node-metastasis (TNM) stage, early recurrence, and shorter survival.

MRI examination using a liver-specific contrast agent enables functional assessment of the liver on hepatobiliary-phase (HBP) images [9]. The “cloud sign” on HBP images, observed as a relatively high cloud-like signal intensity (SI) in the central part of the lesion surrounded by a hypointense peripheral rim, is considered a typical characteristic of IMCC. Pathologically, these characteristics in HBP images represent the center fibrous stroma and the rim area of tumor cells within the tumor [10]. Several studies have shown a relationship between the SI or range of various areas in HBP with the prognoses of patients with IMCC [11, 12]; however, these findings remain controversial. Studies evaluating the relationship between quantitative MRI parameters in various regions of IMCC and their biological characteristics and prognosis are lacking. Therefore, this study was conducted to investigate the value of multimodal quantitative parameters in different areas of the IMCC in the preoperative prediction of aggressiveness and prognosis of patients.

## Materials and methods

### Ethical approval

This retrospective study was approved by the Shandong Provincial Hospital, affiliated to Shandong First Medical University’s Institutional Review Boards (SWYX: No. 2025-160). The requirement for informed consent was waived.

### Patients

**Fig. 1:**
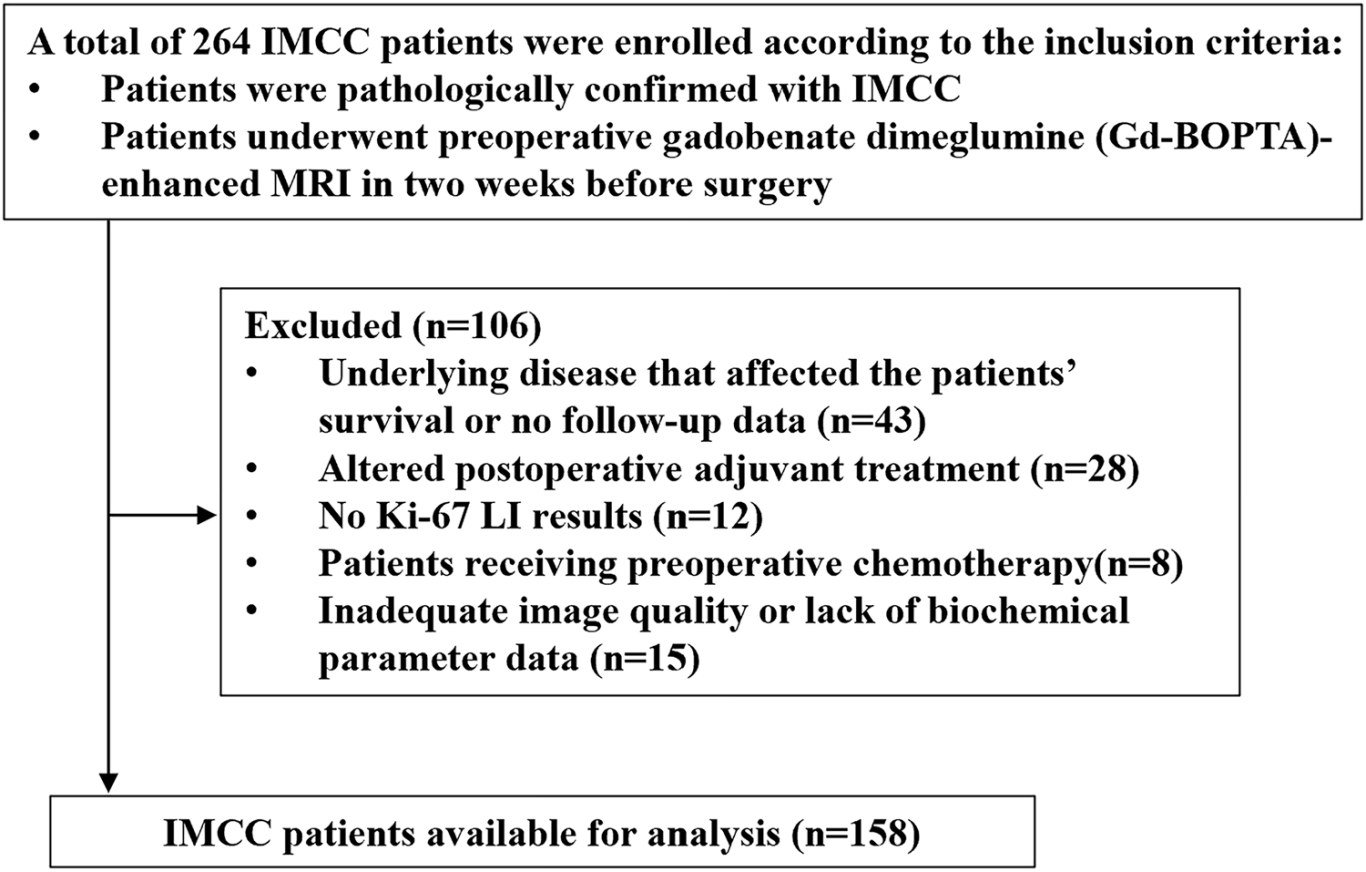
Study flowchart of the enrolled patients. IMCC, intrahepatic mass-forming cholangiocarcinoma; MRI, magnetic resonance imaging; LI, labeling index

The data were retrieved from the electronic medical record databases of three medical institutions from December 2017 to September 2023 (*n* = 264): Shandong Provincial Hospital affiliated to Shandong First Medical University (*n* = 185), The First Affiliated Hospital of Shandong First Medical University (*n* = 46) and Central Hospital Affiliated to Shandong First Medical University (*n* = 33). Patients with IMCC who met the following inclusion criteria were included: (1) patients pathologically confirmed with IMCC, and (2) patients who underwent preoperative gadobenate dimeglumine (Gd-BOPTA)-enhanced MRI at 2 weeks before surgery. The exclusion criteria were as follows: (1) patients with underlying disease impacting patient survival or patients with no follow-up data, such as advanced cardiovascular disease, severe chronic respiratory disease, end‑stage renal disease, decompensated liver cirrhosis, or other terminal illnesses(*n* = 43), (2) patients with altered postoperative adjuvant treatment (*n* = 28), (3) patients with no Ki-67 LI results (*n* = 12), (4) patients receiving preoperative chemotherapy (*n* = 8), and (5) patients with inadequate image quality or lacking biochemical parameter data (*n* = 15). Finally, 158 patients with IMCC were included in this study. The details of patient selection are provided in Fig. 1.

### Clinical data

The preoperative laboratory and clinical data were assessed by reviewing electronic medical records, including data regarding the history of hepatitis B virus serum markers, alpha-fetoprotein, carcinoembryonic antigen, carbohydrate antigen 19-9 (CA19-9), total bilirubin, albumin, prothrombin time, international normalized ratio, aspartate aminotransferase, alanine aminotransferase, and Child-Pugh class. Clinical data were chosen based on published prognostic relevance and data availability [13].

### Histopathological analysis

The histopathological examination was performed by two experienced pathologists (with 15 and 18 years of experience in hepatobiliary pathology, respectively) blinded to radiological and clinical results. In cases of disagreement, a consensus was reached by discussion. The Ki-67 expression level was measured using immunohistochemical staining. The Ki-67 LI was evaluated using the percentage of positive cells in the selected field of view at high power (× 400). As in previous studies [8, 14], patients with IMCC were divided into two groups according to the Ki-67 LI: those with low expression (Ki-67 LI < 25%) and those with high expression (Ki-67 LI ≥ 25%). Moreover, the degree of IMCC differentiation was also evaluated (well or moderately differentiated and poorly differentiated). When a single tumor contained regions of varying degrees of differentiation, the lesion was classified based on the area with the poorest degree of tumor differentiation.

### MRI protocol

All patients were examined on a 3-T MRI scanner. The images from Siemens Prisma and Siemens Magnetom Skyra scanners with a phased-array body coil in the three involved centers were selected. Gd-BOPTA (0.1 mmol/kg bodyweight; Multihance, Bracco) was intravenously injected at a rate of 2 mL/s. Hepatobiliary phase images with Gd-BOPTA were obtained approximately 90 min after intravenous injection. The scan sequence included the following: in-phase and out-of-phase T1-weighted imaging, T2-weighted fat-suppression turbo spin-echo sequence, diffusion-weighted imaging, and dynamic three-dimensional T1-weighted contrast-enhanced imaging. All pulse sequence parameters are shown in Supplementary Material 1.

### Image analysis

MRI scans were independently evaluated by two radiologists (observer 1 and observer 2) with 10 and 20 years of experience in abdominal radiology, respectively. They were blinded to the clinical history of the patients. In case of disagreement, a consensus was reached by discussion. Quantitative analysis was performed by the two radiologists, and the average value was calculated. ITK-SNAP software (ver. 3.6.0, www.itksnap.org) was used for segmenting tumors and measuring volume. The central area was considered the enhancement area of IMCC in HBP images, and the rim area was the peripheral low-SI area. If the lesion showed no “cloud signs” in HBP, the high-signal and low-signal areas were considered as lesions in the central and rim areas, respectively.

**Fig. 2:**
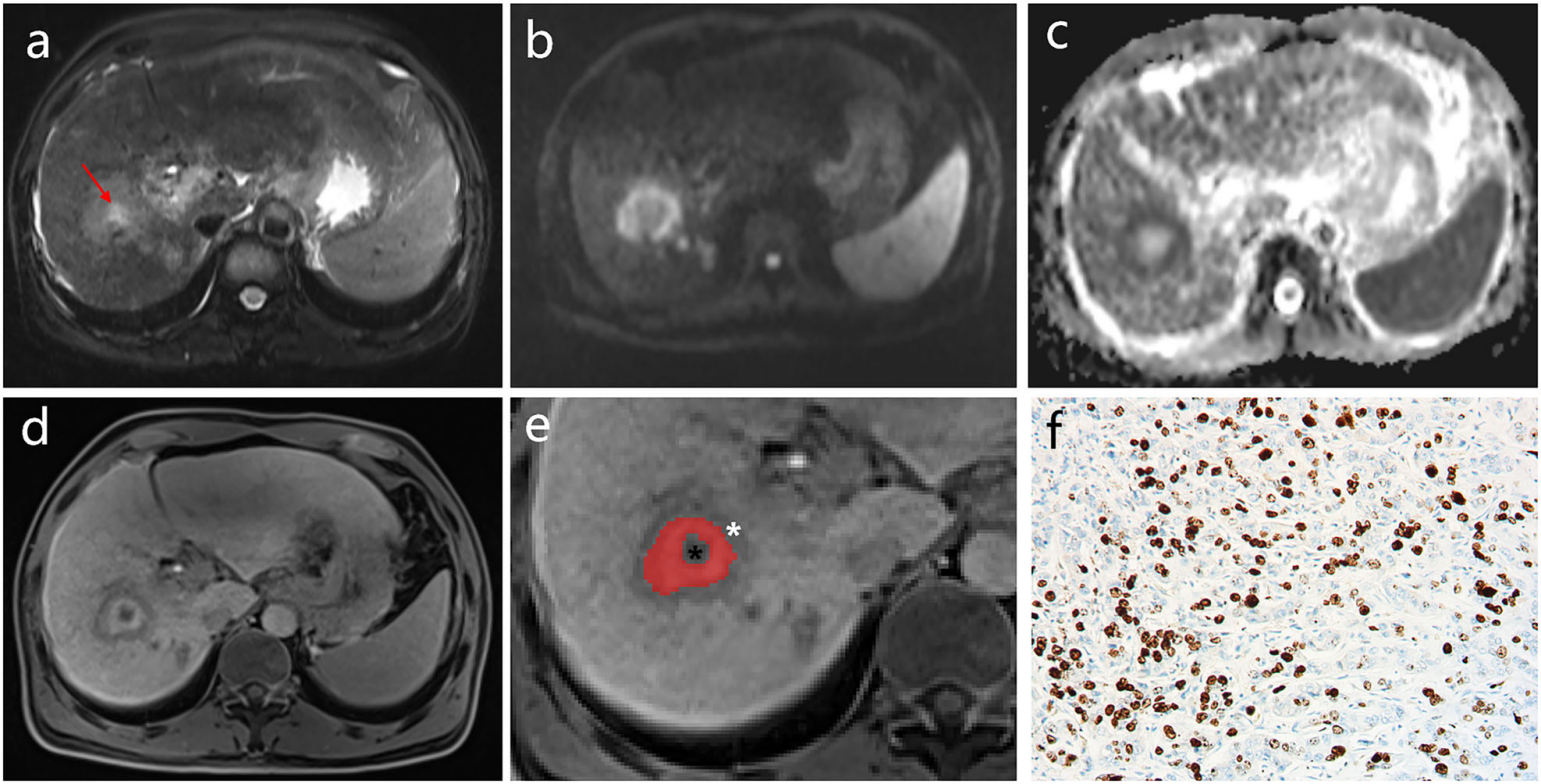
Typical case of high‑aggressiveness IMCC on MRI images. **a**–**e** A 51-year-old male patient with IMCC showed a “targetoid appearance” on MRI. **a** T2WI showed heterogeneous hyperintensity, sometimes with bright signal foci in the center (red arrows). **b** DWI, (**c**) ADC imaging and (**d**) HBP imaging showed a “target sign.” **e** HBP imaging showing the central area (the red region of interest), rim area (white asterisk) and necrosis area (black asterisk) in IMCC. **f** Histopathology showed that the Ki‑67 labeling index of IMCC cells in the high‑aggressiveness group was 60% (immunohistochemical staining, × 400-fold). ADC, apparent diffusion coefficient; DWI, diffusion-weighted imaging; HBP, hepatobiliary phase; IMCC, intrahepatic mass-forming cholangiocarcinoma; MRI, magnetic resonance imaging; T2WI, T2-weighted imaging

**Fig. 3:**
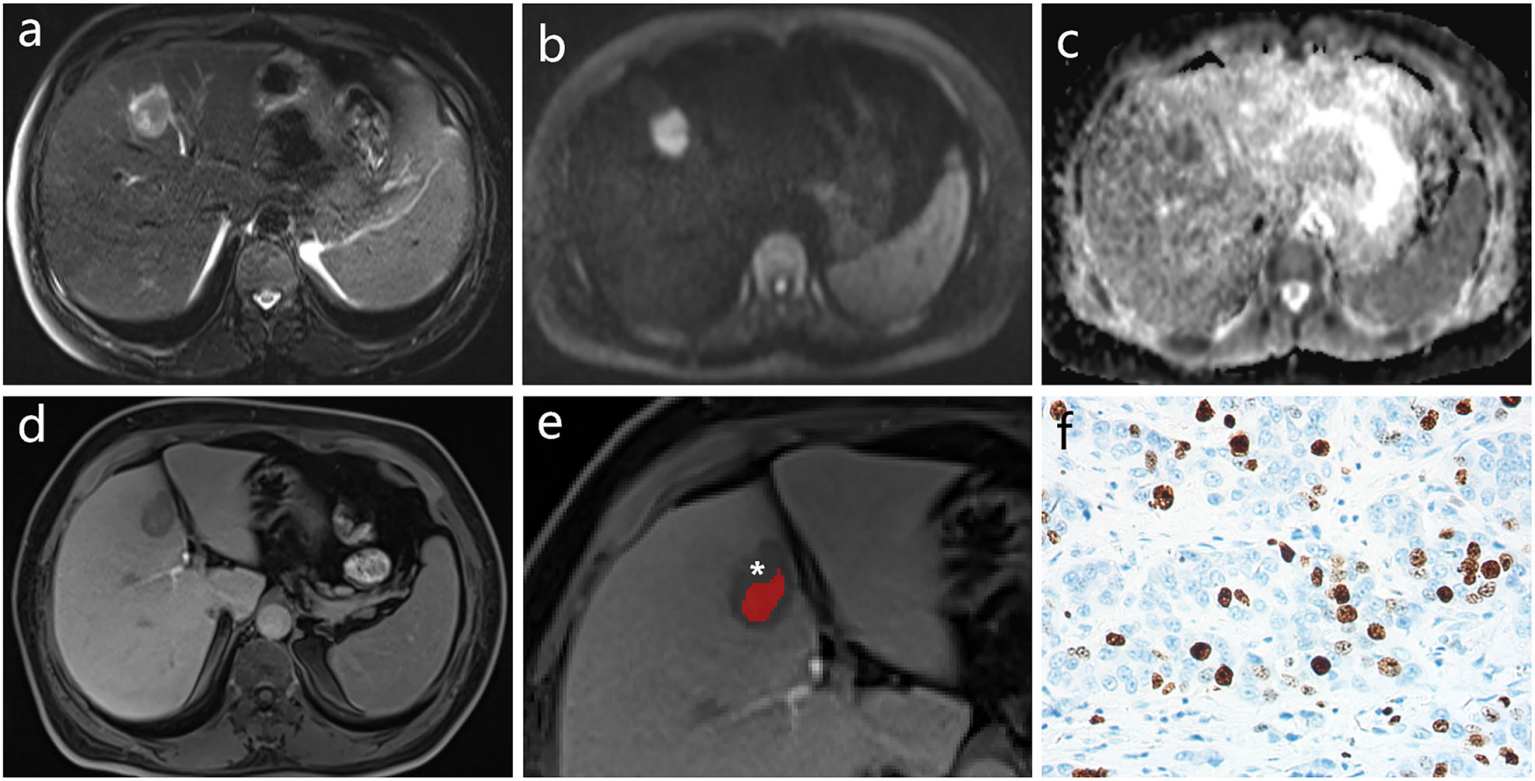
Typical case of low‑aggressiveness IMCC on MRI images. **a**–**e** A 52-year-old female patient with IMCC showed a “targetoid appearance” on MRI. **a** T2WI, (**b**) DWI, (**c**) ADC imaging and (**d**) HBP imaging showed a “target sign.” **e** HBP imaging showing the central area (the red region of interest) and rim area (white asterisk) in IMCC. **f** Histopathology showed that the Ki‑67 labeling index of IMCC cells in the low‑aggressiveness group was 10% (immunohistochemical staining, × 400-fold). ADC, apparent diffusion coefficient; DWI, diffusion-weighted imaging; HBP, hepatobiliary phase; IMCC, intrahepatic mass–forming cholangiocarcinoma; MRI, magnetic resonance imaging; T2WI, T2-weighted imaging

The region of interest (ROI) in the central area and the entire lesion in HBP images were delineated slice by slice within the borders of the tumors. The areas of intratumoral necrosis were not delineated in the central area representing fibrous stroma (the definition of intratumoral necrosis is provided in the qualitative imaging parameters subsection), and then the volume of every area was calculated (*V*_center_ and *V*_tumor_). The volume ratio (VR) of the central area to the entire tumor was defined as follows: VR = 100% × *V*_center_/*V*_tumor_. Also, ROIs were placed in precontrast-phase (PRE) and HBP images to measure SI in the central area (SI_center-HBP_, SI_center-PRE_), rim area (SI_rim-HBP_, SI_rim-PRE_), and liver (SI_liver-HBP_, SI_liver-PRE_). The size and position of each ROI were consistent. The bile ducts, blood vessels, bleeding, cysts, and necrosis were avoided. The ROIs of the lesions were measured three times, and the average was recorded. Based on these quantitative measurements against the background of liver parenchyma, the relative intensity ratio (RIR) of the central and rim areas was defined as follows: RIR_center_ = 100% × SI_center-HBP_/SI_liver-HBP_ and RIR_rim_ = 100% × SI_rim-HBP_/SI_liver-HBP_. The relative enhancement ratio (RER) of the central and rim areas was defined as follows: RER_center_ = 100% × (SI_center-HBP_/SI_liver-HBP_)/(SI_center-PRE_/SI_liver-PRE_) and RER_rim_ = 100% × (SI_rim-HBP_/SI_liver-HBP_)/(SI_rim-PRE_/SI_liver-PRE_) [15]. Furthermore, the mean apparent diffusion coefficient (ADC) values were measured in the liver, tumor center, and rim area (ADC_mean-liver_, ADC_mean-center_, and ADC_mean-rim_) using syngo.via post-processing software. The placement rule of ROI was the same as earlier. The normalized ADC (nADC) values were calculated using ADC values of liver parenchyma as the reference tissue [16, 17]. The nADC of the rim area and the entire tumor was defined as follows: nADC_center_ = ADC_mean-center_/ADC_mean-liver_ and nADC_rim_ = ADC_mean-rim_/ADC_mean-liver_. The measurement process is shown in Figs. 2 and 3.

The qualitative imaging parameters were evaluated as discussed next.

The following MRI characteristics classified into two major categories were recorded for each patient: (1) tumor maximum size (≥ 3 cm/< 3 cm) [18]; (2) tumor boundary (distinct/obscure); (3) arterial peritumoral hyperenhancement (defined as fuzzy-marginated hyperenhancement outside the tumor borders that became isointense with normal liver parenchyma in later dynamic phases) [13]; (4) intrahepatic ductal dilatation: bile duct dilation with diameter ≥ 5 mm; (5) necrosis (defined as bright signal foci on T2-weighted images without contrast enhancement and low-signal foci on the hepatobiliary stage images); (6) liver capsule retraction (present/absent); (g) satellite nodules: small (< 2 cm) tumor nodules around the primary tumor, classified as absent or present [19]; and (7) lymphadenectasis: regional lymph nodes with a short-axis diameter > 1 cm.

### Survival outcomes

Routine postoperative surveillance, including physical examination and laboratory tests, with medical imaging (e.g., computed tomography, ultrasound, or MRI) was conducted every 1–3 months during the first 2 years and every 3–6 months thereafter. The primary endpoints in our study were OS and disease-free survival (DFS). OS was defined as the time from surgery to death, the last follow-up visit, or the date of loss to follow-up. DFS was defined as the time from surgery to follow-up visit providing evidence of tumor progression or death. The survival data were obtained on April 4, 2024.

### Statistical analysis

The inter-reader agreement for any of the features was assessed using the kappa statistic and the intraclass correlation coefficients (ICCs). Quantitative indicators were evaluated using the ICCs, whereas qualitative indicators were analyzed with Cohen’s kappa coefficient. ICCs or Kappa coefficients greater than 0.8 indicated good consistency. For continuous variables, normally distributed data were analyzed using the Student’s *t-*test, whereas non-normally distributed variables were assessed using the Mann–Whitney *U* test. The *χ*^2^ test or Fisher’s exact test was used for categorical variables. The optimal cutoff values were selected using the Youden index. Logistic regression analysis was used to evaluate the risk factors. The discriminating ability was measured using the receiver operating characteristic (ROC) curve and the area under the curve (AUC). The Delong test was applied to compare AUC among various factors. Prognostic factors were evaluated using the Cox proportional-hazards regression model for survival outcomes. Statistical significance was set at *p* ≤ 0.05. Statistical analyses were conducted using SPSS and GraphPad Prism softwares (IBM SPSS Statistics, version 26.0; GraphPad Prism, version 9.2.0).

## Results

### Baseline characteristics

A total of 264 patients were initially screened, of whom 106 were excluded. Finally, 158 patients with IMCC (102 males and 56 females aged 60.7 ± 10.7 years; range 34–81 years) were included in this study. The pathological reevaluation revealed IMCC with high aggressiveness in 104 (65.8%) patients and IMCC with low aggressiveness in 54 (34.2%) patients. The main baseline demographic and clinical characteristics of patients are detailed in Table 1.

**Table 1:** Baseline features of patients with intrahepatic mass-forming cholangiocarcinoma

Characteristic	High aggressive (*n* = 104)	Low aggressive (*n* = 54)	*p*-value
Demographic data			
Age (years)*	62.0 (54.0, 69.3)	58.0 (53.0, 68.0)	0.227
Sex (male/female)	64 (61.5)/40 (38.5)	38 (70.4)/16 (29.6)	0.271
BMI*	23.45 (21.42, 25.90)	23.18 (21.23, 26.05)	0.323
Tumor biomarkers			
AFP (< 20/≥ 20 ng/mL)	86 (82.7)/18 (17.3)	48 (88.9)/6 (11.1)	0.303
CEA (< 5/≥ 5 U/mL)	64 (61.5)/40 (38.5)	42 (77.8)/12 (22.2)	0.686
CA19-9 (< 34/≥ 34 U/mL)	24 (23.1)/80 (76.9)	30 (55.6)/24 (44.4)	< 0.001
Etiology of chronic liver disease			
HBV (N/Y)	83 (79.8)/21 (20.2)	40 (74.1)/14 (25.9)	0.410
Serum markers*			
Albumin (g/L)	38.30 (35.00, 41.62)	41.20 (37.68, 43.90)	0.006
Prothrombin time (s)	12.10 (11.70, 12.68)	12.05 (11.10, 13.13)	0.920
Prothrombin time (INR)	1.05 (0.99, 1.14)	1.07 (1.02, 1.11)	0.770
Total bilirubin (μmol/L)	15.30 (10.63, 21.84)	19.53 (12.82, 64.31)	0.009
Alanine aminotransferase (U/L)	27.50 (17.26, 52.00)	26.00 (22.48, 78.25)	0.083
Aspartate aminotransferase (U/L)	27.50 (20.75, 43.20)	34.00 (24.50, 76.30)	0.012
MRI quantitative factors*			
VR	44.69 (38.00, 54.60)	33.56 (28.36, 37.72)	< 0.001
RIR_center_	75.53 (62.99, 87.98)	91.50 (77.77, 102.90)	< 0.001
RIR_rim_	49.10 (42.64, 60.35)	60.08 (56.64, 75.79)	< 0.001
RER_center_	123.02 (104.45, 150.17)	117.93 (103.29, 131.01)	0.266
RER_rim_	97.90 (81.50, 118.22)	94.30 (80.94, 120.10)	0.955
nADC_rim_	0.78 (0.67, 0.86)	0.78 (0.73, 0.86)	0.782
nADC_center_	1.03 (0.96, 1.16)	1.06 (0.96, 1.34)	0.173
Tumor maximum size	4.36 (3.30, 6.54)	3.55 (2.66, 5.86)	0.018
MRI qualitative factors			
Tumor boundary (distinct/obscure)	36 (34.6)/68 (65.4)	26 (48.1)/28 (51.9)	0.098
Arterial peritumoral hyperenhancement (N/Y)	30 (28.8)/74 (71.2)	13 (24.1)/41 (75.9)	0.523
Satellite nodule (N/Y)	48 (46.2)/54 (53.8)	26 (48.1)/28 (51.9)	0.812
Intrahepatic ductal dilatation (N/Y)	86 (82.7)/18 (17.3)	46 (85.2)/8 (14.8)	0.689
Intratumoral necrosis (N/Y)	44 (42.3)/60 (57.7)	42 (77.8)/12 (22.2)	< 0.001
Liver capsule retraction (N/Y)	86 (82.7)/18 (17.3)	44 (81.5)/10 (18.5)	0.850
Lymphadenectasis (N/Y)	80 (76.9)/24 (23.1)	40 (74.1)/14 (25.9)	0.691
Differentiation (well or moderate/poor)	36 (34.6)/68 (65.4)	20 (37.0)/34 (63.0)	0.763
Child-Pugh grade (A/B/C)	88 (84.6)/16 (15.4)/0 (0.0)	44 (81.5)/10 (17.5)/0 (0.0)	0.614

Unless otherwise stated, data are shown as the number of patients with percentages in parentheses

*BMI* body mass index, *AFP* alpha-fetoprotein, *CEA* carcinoembryonic antigen, *CA19-9* carbohydrate antigen 19-9, *HBV* hepatitis B virus, *INR* international normalized ratio, *MRI* magnetic resonance imaging, *VR* volume ratio, *RIR* relative intensity ratio, *RER* relative enhancement relative, *nADC* normalized apparent diffusion coefficient

* Data are presented as median and interquartile range

### Interobserver agreement

The interobserver agreement for qualitative and quantitative MRI features was good to excellent (*κ* = 0.852–0.930 and ICC = 0.964–0.999) (Supplementary Materials 2 and 3).

### Predictive performance of quantitative parameters

**Fig. 4:**
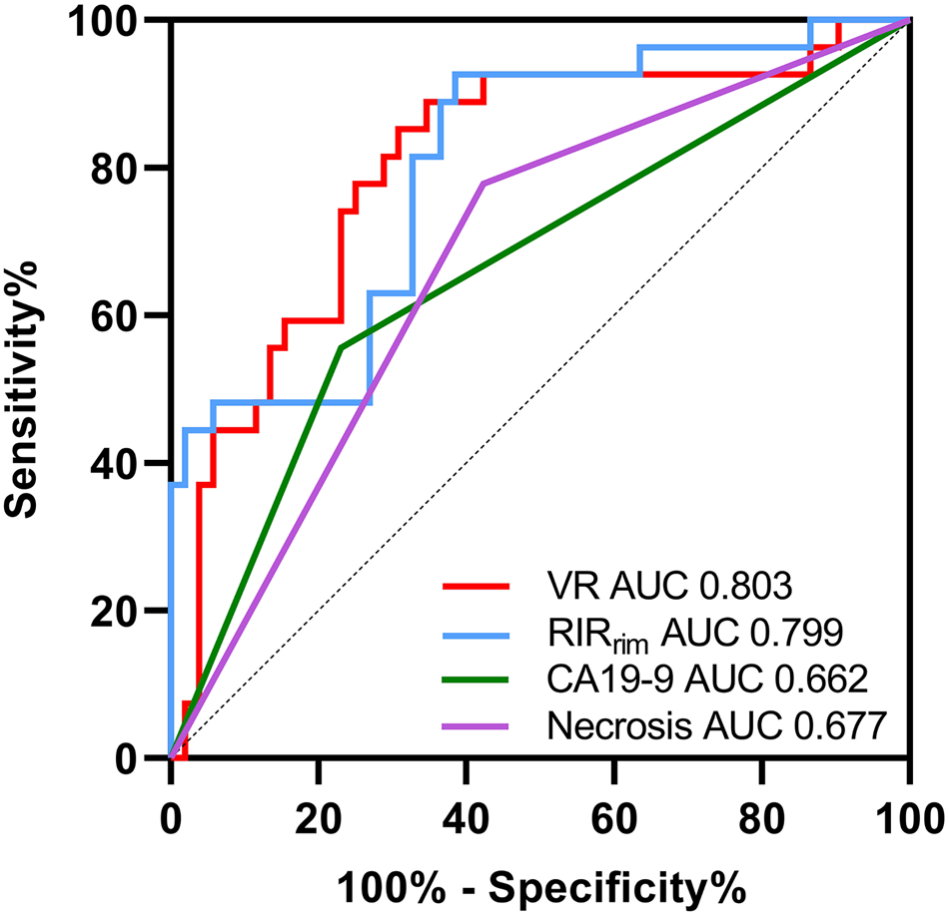
ROC curve comparison of RIR_rim_, VR, CA19-9 ≥ 34 U/mL, and intratumoral necrosis for predicting IMCC with high aggressiveness. AUC, Area under curve; CA19-9, carbohydrate antigen 19-9; IMCC, intrahepatic mass-forming cholangiocarcinoma; RIR, relative intensity ratio; ROC, receiver operating characteristic; VR, volume ratio

Among quantitative MRI parameters, VR had significantly lower values, and RIR_center_ and RIR_rim_ had significantly lower values in the high-aggressiveness group (all, *p* < 0.05). The multivariate logistic regression analysis showed that VR [odds ratio (OR), 1.106; 95% confidence interval (CI), 1.053–1.163; *p* < 0.001], RIR_rim_ (OR, 0.877; 95% CI, 0.833–0.925; *p* < 0.001), CA19-9 ≥ 34 U/mL (OR, 5.724; 95% CI, 2.017–16.245; *p* = 0.001), and necrosis (OR, 4.892; 95% CI, 1.638–14.610; *p* = 0.004) were independent predictors (Table 2). The ROC curve was calculated. VR displayed significantly better diagnostic performance compared with CA19-9 ≥ 34 U/mL (AUC, 0.803 vs AUC, 0.662; *p* = 0.009) and intratumoral necrosis (AUC, 0.803 vs AUC, 0.677; *p* = 0.019) for predicting aggressiveness. Similarly, RIR_rim_ also showed significantly better diagnostic performance compared with CA19-9 ≥ 34 U/mL (AUC, 0.799 vs AUC, 0.662; *p* = 0.011) and necrosis (AUC, 0.799 vs AUC, 0.677; *p* = 0.020) for predicting high aggressiveness (Fig. 4).

**Table 2:** Univariable and multivariable analysis for intrahepatic mass-forming cholangiocarcinoma with high aggressiveness

Variables	Univariable analysis		Multivariable analysis	
	OR (95% CI)	*p*-value	OR (95% CI)	*p*-value
Tumor biomarkers				
CA19-9 (≥ 34 U/mL)	4.167 (2.060, 8.428)	< 0.001	5.724 (2.017, 16.245)	0.001
Serum markers				
Albumin (g/L)	0.972 (0.935, 1.010)	0.142		
Total bilirubin (μmol/L)	0.996 (0.990, 1.002)	0.154		
Aspartate aminotransferase (U/L)	0.999 (0.994, 1.003)	0.527		
MRI quantitative features				
VR	1.105 (1.061, 1.151)	< 0.001	1.106 (1.053, 1.163)	< 0.001
RIR_rim_	0.897 (0.864, 0.932)	< 0.001	0.877 (0.833, 0.925)	< 0.001
RIR_center_	0.994 (0.984, 1.003)	0.184		
MRI qualitative features				
Tumor maximum size ≥ 3 cm	3.285 (1.562, 6.908)	0.002	0.657 (0.125, 3.450)	0.643
Intratumoral necrosis	4.773 (2.254, 10.107)	< 0.001	4.892 (1.638, 14.610)	0.004

*OR* odds ratio, *CI* confidence interval, *CA19-9* carbohydrate antigen 19-9, *MRI* magnetic resonance imaging, *VR* volume ratio, *RIR* relative intensity ratio

### Prognostic value of quantitative parameters for patients with IMCC

During the follow-up period (median, 27 months; range, 1–60 months), 98 of 158 (62.03%) patients with IMCC experienced recurrence during follow-up. The estimated 1-, 2-, and 3-year DFS rates after the surgical resection for IMCC were 63.3%, 43.4%, and 35.4%, respectively. Of the 158 patients with IMCC, 70 (44.3%) died due to IMCC progression. The estimated 1-, 2-, and 3-year OS rates after the surgical resection for IMCC were 82.3%, 66.9%, and 48.8%, respectively.

The univariate and multivariate Cox regression analysis results are presented in Table 3. The multivariate analysis showed that VR [hazard ratio (HR), 1.034; 95% CI, 1.018–1.049; *p* < 0.001], RIR_rim_ (HR, 0.950; 95% CI, 0.926–0.976; *p* < 0.001), CA19-9 ≥ 34 U/mL (HR, 7.337; 95% CI, 2.896–18.591; *p* < 0.001), and tumor boundary (HR, 2.166; 95% CI, 1.184–3.960; *p* = 0.012) were independent predictors of poorer OS. Therefore, VR (HR, 1.035; 95% CI, 1.022–1.049; *p* < 0.001), RIR_rim_ (HR, 0.979; 95% CI, 0.960–0.999; *p* = 0.041), CA19-9 ≥ 34 U/mL (HR, 1.956; 95% CI, 1.102–3.472; *p* = 0.022), tumor boundary (HR, 1.755; 95% CI, 1.058–2.911; *p* = 0.029), and tumor maximum size ≥ 3 cm (HR, 4.383; 95% CI, 1.868–10.288; *p* = 0.001) were independent predictors of shorter DFS.

**Table 3:** Cox analysis of prognosis value for overall survival and disease-free survival in patients with intrahepatic mass-forming cholangiocarcinoma

	OS (overall survival)				DFS (disease-free survival)			
	Univariable analysis		Multivariable analysis		Univariable analysis		Multivariable analysis	
	HR (95% CI)	*p*-value	HR (95% CI)	*p*-value	HR (95% CI)	*p*-value	HR (95% CI)	*p*-value
Age (years)	1.016 (0.994, 1.039)	0.155			1.008 (0.990, 1.027)	0.368		
Sex	1.022 (0.619, 1.685)	0.933			0.823 (0.539, 1.255)	0.365		
BMI	1.000 (0.934, 1.072)	0.991			1.013 (0.955, 1.073)	0.673		
AFP (≥ 20 ng/mL)	1.631 (0.934, 2.851)	0.086			1.437 (0.878, 2.352)	0.150		
CEA (≥ 5 U/mL)	1.139 (0.700, 1.852)	0.600			0.955 (0.629, 1.449)	0.829		
CA19-9 (≥ 34 U/mL)	9.731 (4.186, 22.622)	< 0.001	7.337 (2.896, 18.591)	< 0.001	2.975 (1.837, 4.818)	< 0.001	1.956 (1.102, 3.472)	0.022
HBV	1.064 (0.616, 1.837)	0.825			0.994 (0.618, 1.599)	0.986		
Albumin (g/L)	0.970 (0.935, 1.005)	0.096			0.986 (0.959, 1.014)	0.337		
Prothrombin time (s)	1.187 (0.997, 1.413)	0.053			1.094 (0.934, 1.283)	0.266		
Prothrombin time (INR)	2.953 (0.241, 36.109)	0.397			2.733 (0.349, 21.402)	0.338		
Total bilirubin (μmol/L)	1.003 (0.998, 1.008)	0.246			0.998 (0.993, 1.003)	0.447		
Alanine aminotransferase (U/L)	1.001 (0.999, 1.004)	0.263			0.999 (0.997, 1.002)	0.550		
Aspartate amino-trans-ferase (U/L)	1.002 (1.000, 1.005)	0.103			1.000 (0.997, 1.003)	0.924		
VR	1.042 (1.026, 1.057)	< 0.001	1.034 (1.018, 1.049)	< 0.001	1.038 (1.025, 1.052)	< 0.001	1.035 (1.022, 1.049)	< 0.001
RIR_center_	0.998 (0.989, 1.006)	0.600			0.996 (0.988, 1.004)	0.332		
RIR_rim_	0.948 (0.929, 0.968)	< 0.001	0.950 (0.926, 0.976)	< 0.001	0.964 (0.949, 0.980)	< 0.001	0.979 (0.960, 0.999)	0.041
RER_center_	1.004 (0.999, 1.009)	0.138			1.002 (0.998, 1.007)	0.351		
RER_rim_	1.002 (0.994, 1.010)	0.687			0.999 (0.992, 1.006)	0.782		
nADC_rim_	0.659 (0.087, 5.018)	0.687			1.294 (0.227, 7.359)	0.771		
nADC_center_	0.574 (0.226, 1.457)	0.243			0.527 (0.239, 1.164)	0.113		
Arterial peritumoral hyperenhancement	0.893 (0.527, 1.513)	0.673			1.130 (0.713, 1.791)	0.603		
Tumor maximum size > 3 cm	2.934 (1.404, 6.131)	0.004	1.632 (0.607, 4.386)	0.332	5.524 (2.677, 11.396)	< 0.001	4.383 (1.868, 10.288)	0.001
Tumor boundary	2.352 (1.373, 4.031)	0.002	2.166 (1.184, 3.960)	0.012	1.633 (1.069, 2.495)	0.023	1.755 (1.058, 2.911)	0.029
Satellite nodule	2.933 (1.743, 4.936)	< 0.001	0.810 (0.397, 1.651)	0.562	2.321 (1.535, 3.509)	< 0.001	0.927 (0.544, 1.581)	0.781
Intrahepatic ductal dilatation	1.620 (0.901, 2.911)	0.107			1.226 (0.718, 2.096)	0.455		
Intratumoral necrosis	1.906 (1.185, 3.063)	0.008	0.945 (0.547, 1.633)	0.839	1.938 (1.298, 2.892)	0.001	1.088 (0.705, 1.677)	0.703
Liver capsule retraction	1.007 (0.541, 1.876)	0.982			1.100 (0.659, 1.836)	0.714		
lymphadenectasis	0.665 (0.369, 1.195)	0.172			0.768 (0.474, 1.245)	0.284		

*OS* overall survival, *DFS* disease-free survival, *HR* hazard ratio, *CI* confidence interval, *BMI* body mass index, *AFP* alpha-fetoprotein, *CEA* carcinoembryonic antigen, *CA19-9* carbohydrate antigen 19-9, *HBV* hepatitis B virus, *INR* international normalized ratio, *VR* volume ratio, *RIR* relative intensity ratio, *RER* relative enhancement relative, *nADC* normalized apparent diffusion coefficient

**Fig. 5:**
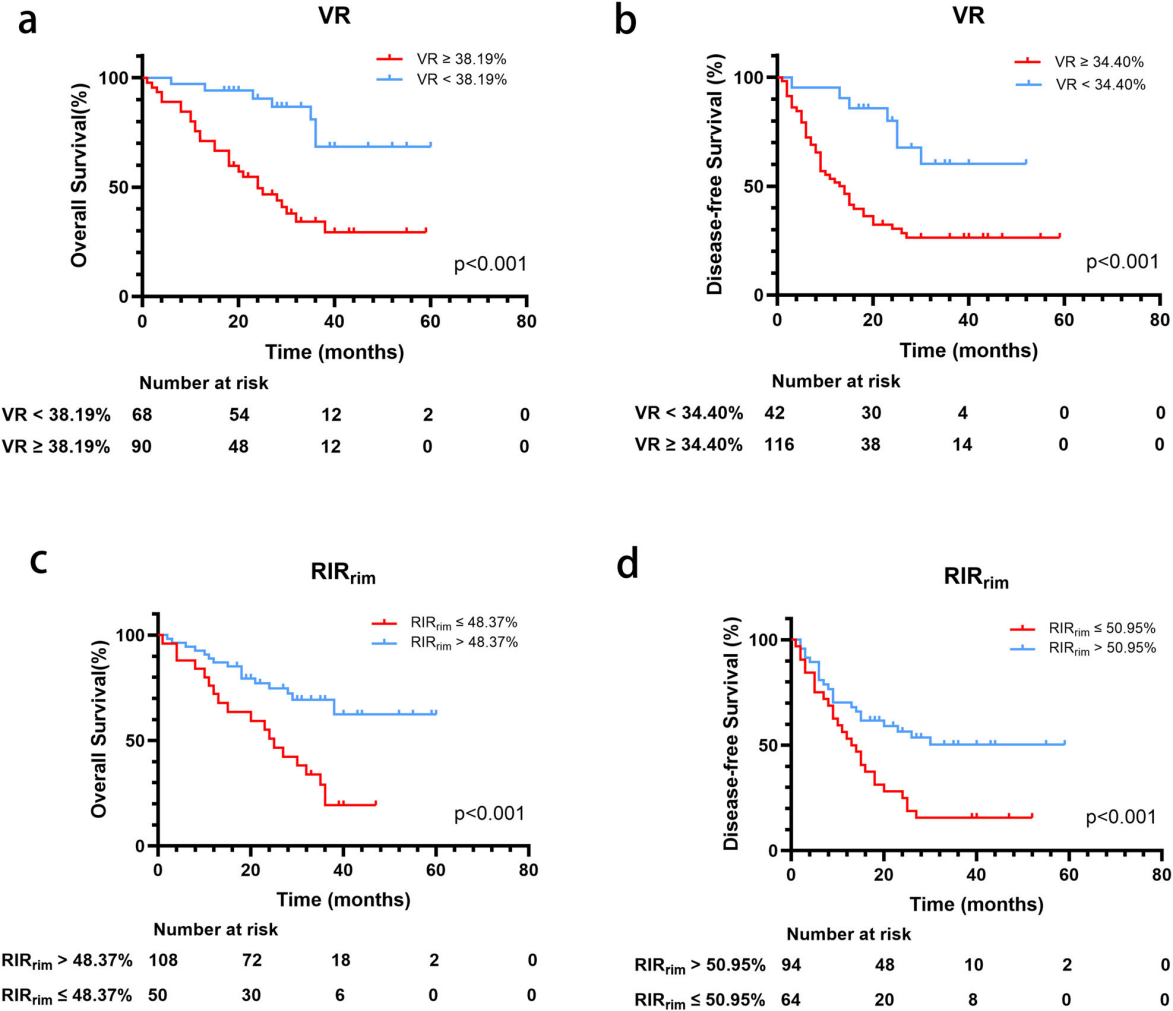
Kaplan–Meier curve to determine the differences in overall survival and disease-free survival. **a** Overall survival comparison between patients with VR ≥ 38.19% and patients with VR < 38.19%. **b** Disease**-**free survival comparison between patients with VR ≥ 34.40% and patients with VR < 34.40%. **c** Overall survival comparison between patients with RIR_rim_ ≤ 48.37% and patients with RIR_rim_ > 48.37%. **d** Disease-free survival comparison between patients with RIR_rim_ ≤ 50.95% and patients with RIR_rim_ > 50.95%. RIR, relative intensity ratio; VR, volume ratio

The most optimized cutoff values for VR and RIR_rim_ to predict OS were 38.19% and 48.37%, respectively. The most optimized cutoff values for VR and RIR_rim_ to predict DFS were 34.40% and 50.95%, respectively. The optimized cutoff values of these parameters were used to plot the Kaplan–Meier survival curves (Fig. 5).

## Discussion

This study evaluated the value of multimodal MRI quantitative parameters for predicting the aggressiveness and prognosis of IMCC. VR and RIR_rim_ were identified as independent predictors of high aggressiveness, OS, and DFS, indicating that noninvasive preoperative MRI could provide a valuable prognostic marker to assist in designing a more rational treatment strategy. Recent studies have shown that gadobenate dimeglumine provides higher diagnostic accuracy than gadoxetate disodium for focal liver lesions, especially small hepatocellular carcinoma, although their interchangeability remains uncertain [20, 21]. Based on these findings, the present study focused specifically on preoperative gadobenate dimeglumine-enhanced MRI features, which may guide surgical planning and reduce unnecessary additional imaging in clinical practice. In view of the uncertain interchangeability and potential differences in enhancement characteristics between the two agents, further studies are warranted to determine whether similar prognostic implications can be observed with gadoxetate disodium.

VR was an independent risk factor for IMCC with high aggressiveness. This was concordant with the findings of Koh et al [11], who qualitatively analyzed the enhancement area of IMCCs on HBP. Their findings indicated that greater enhancement in HBP was correlated with a more fibrous stroma and a poor prognosis. However, qualitative parameters are more subjective than quantitative parameters because they depend on the subjective judgment of the observer. Our study calculated the VR of the central areas of IMCC in the HBP of the entire tumor. The results showed that the central enhancement area of IMCC reflected the fibrous stroma and was strongly associated with higher tumor aggressiveness, resulting in a poorer prognosis. Also, Min et al [22] reported that the area of arterial enhancement was usually considered tumor tissue, whereas the other part likely reflected necrosis or stromal desmoplasia.

Furthermore, this study pioneered the application of RIR_rim_ for predicting IMCC aggressiveness. The results demonstrated a negative correlation of RIR_rim_ with aggressiveness. This was consistent with the findings of Zheng et al [15], who suggested the best predictive performance of RIR in predicting the aggressiveness of hepatocellular carcinoma compared with other quantitative parameters of HBP. Normal hepatocytes take up the contrast agent via organic anion transporting polypeptides (OATP) and excrete it through the biliary system. The expression of OATP1B1/B3 usually decreases (or is absent) without or with partially functioning hepatocytes.

OATP1B3 has been identified as the predominant transporter; its expression level significantly positively correlates with the enhancement intensity observed in the hepatobiliary phase of contrast-enhanced MRI [23, 24]. The decreased RIR_rim_ of IMCC reflected tumor-induced loss of normal hepatocytes. This led to a decrease in the expression of OATP, thus resulting in less uptake of Gd-BOTPA. However, the association between Ki-67 LI and OATP may need further investigation.

ADC values can be used to assess tumor aggressiveness, subtype, and prognosis in the clinic [25]. Hokkoku et al [17] demonstrated a relationship between the lower mean and minimum ADC of IMCC with high Ki-67 expression; the minimum ADC value could more accurately reflect the tumor region. However, we did not observe a significant difference in nADC_center_ and nADC_rim_ between the high- and low-Ki-67 groups, which might be due to the differences in the differentiation degrees of IMCC tumors. ADC values may be influenced by acquisition parameters, susceptibility or motion artifacts, and ROI placement, all of which can introduce measurement bias. In this study, we minimized these effects by using a standardized protocol, applying breath-hold or respiratory-triggered imaging, and placing ROIs consistently on the largest lesion cross-section while avoiding necrotic, vascular, and biliary areas. Nevertheless, variability cannot be entirely excluded. Future studies should further standardize parameters, perform repeated multi-observer measurements, and explore advanced diffusion techniques such as diffusion tensor imaging and diffusion kurtosis imaging to provide more comprehensive microstructural information beyond conventional ADC.

Necrosis was found to be an independent predictor of high Ki-67 LI. This was consistent with the conclusion of previous studies [12, 16], which considered that the high-expression Ki-67 group with less differentiated IMCC might contribute to more tumor necrosis and cause hypointensity in HBP. However, no significant difference in differentiation degree was noted between the high- and low-aggressiveness groups in our study. This might be because only patients with “target appearance” were included in our study. Elevated CA19-9 level was more common in the high-aggressiveness group, which was consistent with the conclusions of Gao et al [13]. This is widely accepted as a useful diagnostic and prognostic biomarker in patients with biliary-pancreatic tumors; an elevated level of CA19-9 is strongly correlated with poor tumor prognosis [26, 27].

This study identified significant differences in albumin, total bilirubin, and aspartate aminotransferase levels across Ki-67 expression groups in the univariate analysis, but no statistically significant difference in the multivariate analysis. Although previous studies demonstrated strong predictive performance of prognostic models integrating these parameters in intrahepatic cholangiocarcinoma [28], the associations observed in this study might be partially obscured by confounding factors such as tumor stage and underlying liver conditions. These findings suggested that the impact of liver function parameters on tumor proliferative activity may be better reflected through comprehensive evaluation systems rather than their independent effects alone.

The survival analysis showed that the VR and RIR_rim_ were independent predictors of OS and DFS in patients with IMCC who underwent surgical resection. The association between these parameters and Ki-67 expression might explain the result. Higher Ki-67 expression is related to higher aggressiveness, resulting in early recurrence and poor prognoses for patients with IMCC. Patients with VR ≥ 38.19% or RIR_rim_ < 48.37% had poorer OS, and patients with VR ≥ 34.40% or RIR_rim_ < 50.95% were associated with shorter DFS. Early detection of IMCCs using VR and RIR_rim_ could help improve the prognosis of these patients. Tumor boundary was identified as an independent risk factor for OS and DFS in patients with IMCC, consistent with previous reports [29–31]. This could be explained by the microinvasion and fewer lymphatic infiltrating cells of the tumor. Moreover, increased CA19-9 level was an independent risk factor for OS and DFS in patients with IMCC, whereas tumor maximum size ≥ 3 cm was an independent risk factor for DFS, which was consistent with previous findings [12, 32–34]. Satellite nodules were significantly associated with shorter OS and DFS in the univariate analysis, similar to the findings of Tang et al [35].

This study had several limitations. First, this was a retrospective study, with a long inclusion time. Also, the rare incidence of IMCC resulted in a small number of patients, and several well-established prognostic factors (surgical margin status, vascular/perineural invasion, adjuvant therapy) were unavailable or excluded because of incomplete documentation, potentially confounding the associations observed between MRI biomarkers and outcome. Second, the study included only patients with typical IMCC who underwent surgery, and therefore, the results may not fully apply to other treatments. Third, matching MRI scans of specific tumors with histopathological results proved to be challenging. Fortunately, previous studies provided evidence supporting the histopathological interpretation of IMCC signs. Additionally, the reported ROC and Cox analyses were limited to univariate associations, and the absence of a combined multivariable prognostic model, calibration analysis, and decision-curve evaluation limits the assessment of overall predictive performance and clinical applicability. Thus, future studies should involve larger sample sizes and validation with a composite prognostic model to confirm these results, including patients with IMCC and other MRI manifestations in HBP.

## Conclusion

VR and RIR_rim_ derived from Gd-BOPTA-enhanced MRI had clinical significance for predicting high tumor aggressiveness and prognoses in patients with IMCC.

## ELECTRONIC SUPPLEMENTARY MATERIAL

Supplementary information

## Data Availability

The datasets used and/or analyzed during the current study are available from the corresponding author upon reasonable request.

## Abbreviations

AUC: Area under the curve
CA19-9: Carbohydrate antigen 19-9
CI: Confidence interval
DFS: Disease-free survival
Gd-BOPTA: gadobenate dimeglumine
HBP: Hepatobiliary phase
HR: Hazard ratio
ICC: Intraclass correlation coefficient
IMCC: Intrahepatic mass-forming cholangiocarcinoma
LI: Labeling index
MRI: Magnetic resonance imaging
nADC: Normalized apparent diffusion coefficient
OATP: Organic anion-transporting polypeptides
OR: Odds ratio
OS: Overall survival
RER: Relative enhancement ratio
RIR: Relative intensity ratio
ROC: Receiver operating characteristic
ROI: Region of interest
SI: Signal intensity
VR: Volume ratio
