# Supplementary material for: The value of gadobenate dimeglumine-enhanced MRI quantification in predicting aggressiveness and prognosis of typical intrahepatic mass-forming cholangiocarcinoma: a multicenter retrospective study
Source: Insights Imaging. 2026 Mar 3;17:61. doi: 10.1186/s13244-026-02225-4 (PMC12957741; doi:10.1186/s13244-026-02225-4)
Supplement: Supplementary file 1 — Supplementary information [file 13244_2026_2225_MOESM1_ESM.pdf]

The value of gadobenate dimeglumine-enhanced MRI quantification in predicting aggressiveness and prognosis of typical intrahepatic mass-forming cholangiocarcinoma: A multicenter retrospective study

ELECTRONIC SUPPLEMENTARY MATERIAL

Supplementary Material 1: MRI protocols and detailed parameters of the MRI sequences

MR images were acquired using a 3.0-T MRI scanner (Magnetom Verio and Magnetom Skyra; Siemens Healthcare, Erlangen, Germany) with a 16-channel phase-array coil that covered the whole liver. The MR scan sequences were as follows: (1) a three-dimensional volume-interpolated breath-hold examination (3D VIBE) T1-weighted in-phase with the time of repetition (TR) = 4.16 ms, time of echo (TE) = 2.58 ms, field of view (FOV) = 26 × 32 cm^2, and slice thickness = 5 mm; (2) a 3D VIBE T1-weighted out-phase with TR = 4.16 ms, TE = 1.35 ms, FOV = 26 × 32 cm^2, and slice thickness = 5 mm; (3) a respiration-triggered T2-weighted fat-suppression turbo spin-echo with TR = 3.920–7.345 ms, TE = 105 ms, FOV = 26 × 32 cm^2, and slice thickness = 6 mm; (4) diffusion-weighted imaging (DWI, b = 0 or 50, 800 s/mm^2) with a free-breathing single-shot echo-planar technique and TR = 5.300 ms, TE = 57 ms, FOV = 26 × 32 cm^2, and slice thickness = 8 mm.

A food fasting of more than 6 h and a water fasting of more than 4 h before scanning were required for all patients. A dose of 0.1 mmol per kg of bodyweight of gadobenate dimeglumine (Gd-BOPTA, MultiHance, Bracco Imaging) was injected as a rapid bolus, immediately followed by the administration of 30 mL of saline at a rate of 1 mL/s. The dynamic enhancement was also performed with the 3D VIBE T1-weighted imaging fat saturation sequence (arterial phase, late arterial phase, portal venous phase, and hepatobiliary phase) before and after contrast injection. The images in the arterial phase, later arterial phases, and portal venous phases were obtained during suspended respiration in 20, 50, and 80 s, respectively. All patients rested for 1 h. Subsequently, hepatobiliary-phase (90 min) imaging was performed. The 3D T1-VIBE parameters were as follows: TR = 3.31 ms; TE = 1.3 ms; number of partitions = 72; slice thickness = 3 mm; flip angle = 9°; acceleration factor = 1; FOV = 380 × 308 mm^2; bandwidth = 450 Hz/pixel; matrix = 182 × 320; and acquisition time = 17 s.

Supplementary Material 2

Kappa values between two observers in the whole cohort for the qualitative MRI parameters

Parameters	Kappa value (95% CI)	Approximate significance
Tumor boundary	0.921 (0.859, 0.983)	<0.001
Arterial peritumoral hyperenhancement	0.892 (0.814, 0.970)	<0.001
Intrahepatic ductal dilatation	0.930 (0.851, 1.000)	<0.001
Liver capsule retraction	0.850 (0.742, 0.959)	<0.001
Necrosis	0.924 (0.864, 0.984)	<0.001
Lymphadenectasis	0.882 (0.796, 0.967)	<0.001
Satellite nodules	0.911 (0.847, 0.976)	<0.001

CI, Confidence interval; MRI, magnetic resonance imaging.

Supplementary Material 3

Interclass correlation coefficient for quantitative MRI parameters

Parameters	Method	ICC (95% CI)	Approximate significance
tumor maximum size	Single measures	0.998 (0.998, 0.999)	<0.001
	Average measures	0.999 (0.999, 0.999)	<0.001
VR	Single measures	0.972 (0.962, 0.980)	<0.001
	Average measures	0.986 (0.981, 0.990)	<0.001
RIR_{center}	Single measures	0.996 (0.995, 0.997)	<0.001
	Average measures	0.998 (0.997, 0.999)	<0.001
RIR_{rim}	Single measures	0.996 (0.995, 0.997)	<0.001
	Average measures	0.998 (0.998, 0.999)	<0.001
RER_{center}	Single measures	0.997 (0.995, 0.998)	<0.001
	Average measures	0.998 (0.998, 0.999)	<0.001
RER_{rim}	Single measures	0.996 (0.994, 0.997)	<0.001
	Average measures	0.998 (0.997, 0.998)	<0.001
$nADC_{center}$	Single measures	0.993 (0.990, 0.995)	<0.001
	Average measures	0.997 (0.995, 0.997)	<0.001
$nADC_{rim}$	Single measures	0.964 (0.951, 0.974)	<0.001
	Average measures	0.982 (0.975, 0.987)	<0.001

CI, Confidence interval; ICC, interclass correlation coefficient; nADC, normalized apparent diffusion coefficient; MRI, magnetic resonance imaging; RER, relative enhancement ratio; RIR, relative intensity ratio; VR, volume ratio.
